# Using a Portable Active Sensor to Monitor Growth Parameters and Predict Grain Yield of Winter Wheat

**DOI:** 10.3390/s19051108

**Published:** 2019-03-05

**Authors:** Jiayi Zhang, Xia Liu, Yan Liang, Qiang Cao, Yongchao Tian, Yan Zhu, Weixing Cao, Xiaojun Liu

**Affiliations:** 1 National Engineering and Technology Center for Information Agriculture, Nanjing Agricultural University, Nanjing 210095, China; 2017101023@njau.edu.cn (J.Z.); liuxia224466@163.com (X.L.); 2016101028@njau.edu.cn (Y.L.); qiangcao@njau.edu.cn (Q.C.); yctian@njau.edu.cn (Y.T.); yanzhu@njau.edu.cn (Y.Z.); caow@njau.edu.cn (W.C.); 2 Key Laboratory for Crop System Analysis and Decision Making, Ministry of Agriculture and Rural Affairs, Nanjing Agricultural University, Nanjing 210095, China; 3 Jiangsu Key Laboratory for Information Agriculture, Nanjing Agricultural University, Nanjing 210095, China; 4 Jiangsu Collaborative Innovation Center for Modern Crop Production, Nanjing Agricultural University, Nanjing 210095, China

**Keywords:** active sensor, winter wheat, vegetation index, growth parameters, grain yield

## Abstract

Rapid and effective acquisition of crop growth information is a crucial step of precision agriculture for making in-season management decisions. Active canopy sensor GreenSeeker (Trimble Navigation Limited, Sunnyvale, CA, USA) is a portable device commonly used for non-destructively obtaining crop growth information. This study intended to expand the applicability of GreenSeeker in monitoring growth status and predicting grain yield of winter wheat (*Triticum aestivum* L.). Four field experiments with multiple wheat cultivars and N treatments were conducted during 2013–2015 for obtaining canopy normalized difference vegetation index (NDVI) and ratio vegetation index (RVI) synchronized with four agronomic parameters: leaf area index (LAI), leaf dry matter (LDM), leaf nitrogen concentration (LNC), and leaf nitrogen accumulation (LNA). Duration models based on NDVI and RVI were developed to monitor these parameters, which indicated that NDVI and RVI explained 80%, 68–70%, 10–12%, and 67–73% of the variability in LAI, LDM, LNC and LNA, respectively. According to the validation results, the relative root mean square error (RRMSE) were all <0.24 and the relative error (RE) were all <23%. Considering the variation among different wheat cultivars, the newly normalized vegetation indices rNDVI (NDVI vs. the NDVI for the highest N rate) and rRVI (RVI vs. the RVI for the highest N rate) were calculated to predict the relative grain yield (RY, the yield vs. the yield for the highest N rate). rNDVI and rRVI explained 77–85% of the variability in RY, the RRMSEs were both <0.13 and the REs were both <6.3%. The result demonstrates the feasibility of monitoring growth parameters and predicting grain yield of winter wheat with portable GreenSeeker sensor.

## 1. Introduction

Wheat (*Triticum aestivum* L.) is increasingly important in consequence of its role as a staple calories output, in particular for the Chinese population [1,2]. Due to a further growing population with a constant or even decreasing planting area, crop cultivation management aiming at high production and sustainability of natural resources is required. Narrowing the gap between potential and current yield in developed and developing countries is the main goal for modern crop production [3]. Therefore, accurately monitoring crop growth status based on remote sensing and proximal sensing should be an effective technical approach for improving economic benefits and reducing environmental pollution [4,5]. 

Leaf area index (LAI), dry matter, and nitrogen (N) are the main growth indicators for crop growth status monitoring and yield prediction [6,7,8]. However, the traditional destructive methods for measuring biophysical and biochemical parameters of crop are laborious and time consuming. Among various indirect methods for measuring plant N nutrient status, chlorophyll meter is most widely used [9]. Yuan et al. [10] established prediction models of plant nitrogen accumulation and nitrogen nutrition index using chlorophyll meter values. In 2018, Padilla et al. [9] concluded that chlorophyll meters are suitable for on-farm use to provide rapid assessment of crop N status. Nevertheless, the chlorophyll content is measured only on a single point of leaf, and N concentrations are not homogeneous either within a leaf [11] or crop canopy [12]. Therefore, canopy remote sensing provides new chance for monitoring crop growth and nutrition status.

Remote sensing has been widely applied for crop production management, rapidly and non-destructively monitoring crop growth and N status at small- or large-scale application by using spectral vegetation indices [13,14]. However, satellite-based measurements are often limited by cloudy weather, low temporal and spatial resolution, and satellite remote sensing data is difficult to obtain for common farmers. Unmanned aerial vehicles (UAVs) based remote sensing is a promising approach to overcome the limitations of ground and satellite remote sensing; it is also a promising alternative for precision crop management [15,16,17]. Nonetheless, such systems are still new and mainly used in research domain, with several challenges to overcome [15,16]. In the meantime, active canopy sensors (ACS), unrestricted by weather conditions, remain an important tool for monitoring crop growth and N status. Active canopy multispectral sensors are highly suitable for site-specific crop management (SSCM), comparing to passive sensors easily influenced by environmental conditions and hyperspectral sensors with high price [18,19]. GreenSeeker (Trimble Navigation Limited. Sunnyvale, CA, USA) is an active crop canopy sensor with two wavelengths (red, 671 nm and near-infrared (NIR), 780 nm), which is widely used to monitor crop growth and nutrient conditions in recent years. Osborne [20] used GreenSeeker to estimate growth and N status in spring wheat, and found that the NDVI significantly correlated with biomass, N concentration and plant N uptake as well as grain yield. Cao et al. [21] found that GreenSeeker-NDVI was exponentially related to N uptake in winter wheat, whereas the correlation between N uptake and RVI was linear.

Vegetation index, like normalized difference vegetation index (NDVI) and ratio vegetation index (RVI), can help enhance the interpretation ability of remote sensing data, and has been widely used as a remote sensing means in land use cover detection [22], vegetation cover density evaluation [23], crop identification [24] and crop growth monitoring [25,26,27]. Among various vegetation indices, NDVI and RVI have been proposed and used for decades, and were most widely used in monitoring plant chlorophyll [28], N content [26], and disease [29,30]. In yield prediction, Lopresti et al. [31] developed the relationship between the moderate-resolution imaging spectroradiometer (MODIS) NDVI data and wheat yield. He et al. [32] studied on double-cropping rice in Southern China and found that the relative yield estimation model based on integral vegetation index derived from canopy sensors could accurately predict grain yield. In general, crop growth indices and yield prediction models have been established based on vegetation indices, but these models varied greatly in different crops and production regions, and the reliability and usability of the model are also affected by different types of sensors. Moreover, yield variability is even greater under different varieties and eco-sites, and the yield prediction models based on traditional vegetation index appears more uncertain [33]. 

Therefore, the objectives of this study were: (1) to quantitate relationships of growth parameters (e.g., LAI, LDM, LNC, LNA) with canopy vegetation index in winter wheat, and (2) to calibrate optimal model based on newly normalized vegetation index for predicting grain yield in wheat-rice cropping area of China. This study will provide a technical support for sensor-based crop growth status monitoring and promote the application of remote sensing technology in practical agricultural production.

## 2. Materials and Methods

### 2.1. Experimental Design

**Figure 1:**
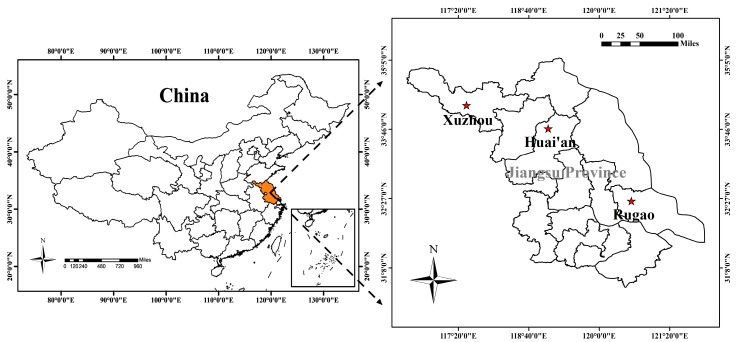
Study sites: wheat experiments conducted at Rugao, Xuzhou, and Huai’an Experimental Station in Jiangsu province of China.

Four field experiments were conducted with different N application rates (0–375 kg·ha^−1^) [34] and varieties at Rugao Experimental Station (32°27′ N, 120°76′ E), Xuzhou Experimental Station (34°48′ N, 117°13′ E) and Huai’an Experimental Station (33°60′ N, 118°88′ E) in Jiangsu province of China, as shown in Figure 1, detailed information about four experiments was summarized in Table 1. A randomized complete block design with three replications was used in all experiments. The N fertilizer used was urea with 46% N. The distribution of total N before sowing and at stem elongation was 50% and 50%. Phosphorus and potassium fertilizers, as monocalcium phosphate Ca(H_2_PO_4_)_2_ and potassium chloride (KCl), were applied before sowing at rates of 135 kg·ha^−1^ (P_2_O_5_) and 190 kg·ha^−1^ (K_2_O). Among the four field trials, experiments 1–3 provided the calibration dataset and experiment 4 served as the validation dataset.

### 2.2. Sample Collection and Measurement

Wheat canopy spectra was measured using a handheld GreenSeeker^®^ Model 505 (Trimble Navigation Limited, Sunnyvale, CA, USA) active optical sensor, which included near-infrared (780 ± 6 nm) and red light (671 ± 6 nm). All measurements were taken on sunny days and no wind or breeze; the carried sensor probe was passed over the crop at a height of approximately 0.8 m above wheat canopy. The sensor path was parallel to the seed rows with the beam of light being perpendicular to the seed row. Each cell consisted of three rows, and each row had the measurement of five replications, with the average values used to represent each plot.

As soon as the canopy reflectance was collected, sampling was carried out synchronously. Detailed sampling dates for each experiment were shown in Table 1. In order to examine the performance at different growth stages, the data were classified into two groups based on Feekes growth stages 4–7 (before canopy closure) and 8–10 (after canopy closure) [35]. Fresh plants were collected from each plot and then separated into green leaf blades (leaves) and culm plus sheath (stems), green leaf blades were used to determine LAI with a LI-3000 portable area meter (Li-Cor, Lincoln, NE, USA), then all of the samples were heated for 30 min at 105 °C to halt metabolic processes, and dried at 80 °C in a forced-draft oven until a constant weight was reached. After dry matter was determined, samples were ground in a Wiley mill, passed through a 1 mm sieve, stored in plastic bags at room temperature until further chemical analysis. Each sample with 0.2 g weight were digested to determine the N concentration using a continuous-flow auto-analyzer (Bran + Luebbe, Hamburg, Germany). 

Grain yield was determined by harvesting plants manually for a 2 m^2^ area in each plot and adjusting to a moisture content of 12.5%.

### 2.3. Data Processing and Analysis

#### 2.3.1. Spectral Data 

Two commonly used spectral indices were determined in this study: NDVI Equation (1) and RVI Equation (2), and the measurements were based on the average spectral value of each plot:

(1) NDVI=(NIR−R)/(NIR+R)

(2) RVI=NIR/R

where NIR is the reflectance of near infrared wave band, R is the reflectance of red band.

#### 2.3.2. N Indicators

For the N indicators, LDM (t·ha^−1^) was used to represent dry matter of canopy leaves and LNC (N%LDM) represent N concentration of canopy leaves. LDM and LNC were multiplied to calculate leaf N accumulation (LNA, kg·ha^−1^) during a specific growth stage [36], as shown in Equation (3):

(3) LNA=LDM×LNC×1000

#### 2.3.3. Relative Grain Yield

In order to minimize the spectra variation among different wheat cultivars and eco-sties, relative NDVI (rNDVI, Equation (4)) [37] and relative RVI (rRVI, Equation (5)) were calculated to predict the relative grain yield (RY, Equation (6)):

(4) $$rNDVI=NDVI_{i}/NDVI_{\max}$$

(5) $$rRVI=RVI_{i}/RVI_{\max}$$

(6) $$RY=Y_{i}/Y_{\max}$$

where NDVI_i_ and RVI_i_ are the measured NDVI and RVI, NDVI_max_ and RVI_max_ represent the measured NDVI and RVI values of the highest N rate plots. Y_i_ is the measured grain yield, Y_max_ represents the grain yield of the highest N rate plots. 

#### 2.3.4. Model Construction and Evaluation

The datasets from Experiments 1–3 (Table 1) were used to construct monitoring models, irrespective of the year, eco-site and cultivar, and the models were calibrated using the dataset from Experiment 4, R^2^, relative error (RE) and relative root mean square error (RRMSE; Equation (7)) were used in the model construction and calibration with Origin 2018 Pro software (Origin Lab Corporation, Northampton, MA, USA). Curves were drawn with Origin 2018 Pro software. The analysis of variance (ANOVA) was done using IBM SPSS 25 software (IBM Corporation, Armonk, NY, USA).

(7) $$RRMSE(\%)=\sqrt{\frac{{\sum_{i=1}^{n}\left(P_{i}-O_{i}\right)}^{2}}{n}}\times \frac{100}{\bar{O_{i}}}$$

where n is the number of samples, O_i_ is the observed value, P_i_ is the predicted value derived from the model, and ${\bar{O}}_{i}$ is the mean observed value.

## 3. Results

### 3.1. Variation in Agronomic Parameters

The LAI in the calibration dataset ranged from 1.16 to 9.47 (CV = 49.24%) across growth stages and site-years (Table 2), while LDM was 0.56–3.90 t·ha^−1^ (CV = 41.86%) (Table 2), and LNC was 17.52–43.12 g·kg^−1^ (CV = 18.56%) (Table 2), LNA was 14.99–142.37 kg·ha^−1^ (CV = 48.66%) (Table 2). The analysis showed that LAI and LDM were more variable during Feekes growth stages 4–7 (CV = 43.02% and 39.44%, respectively) than stages 8–10 (CV = 38.97% and 34.16%, respectively), and LNC showed more variable during Feekes stages 8–10 (CV = 20.66%) than stages 4–7 (CV = 15.97%), while LNA during the two stages was similar (CV = 43.92% and 44.88%, respectively) (Table 2). In the analysis of variance, varieties had no significant influence on four agronomic parameters, while the treatment of N rates was significant (Table 3). Years had no significant influence on LAI and LNC, but significant influence on LDM and LNA, this may be caused by manual random sampling error (Table 3). The interaction of N rates and years, N rates and varieties had no significant effect on each parameter (Table 3). These results indicated that wheat growth was significantly affected by N application rate; in addition, the large variability in N-related parameters establishes the suitability of the dataset for evaluating the performance of GreenSeeker sensor.

### 3.2. Estimating Leaf Area Index

**Figure 2:**
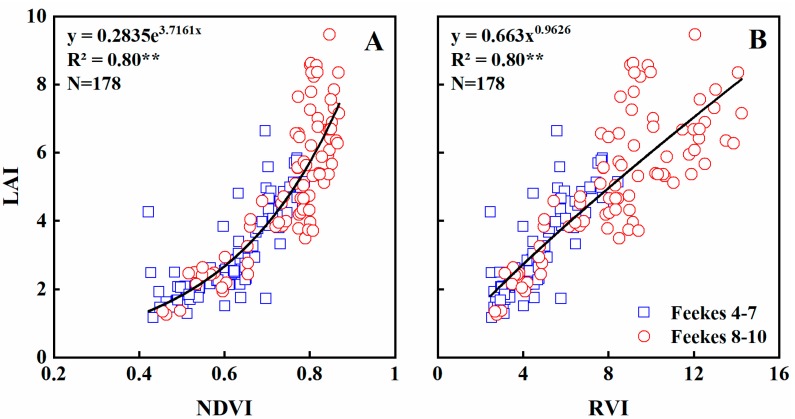
Quantitative relationships of NDVI (**A**) and RVI (**B**) to LAI in wheat varieties under varied N rates across all growth stages.

Crop growth indicators, e.g., LAI, reflect main biophysical processes and growth status [38]. Experimental data indicate that NDVI was exponentially related to LAI (R^2^ = 0.80; *p* < 0.01) across all eco-sites and growth stages (Figure 2A). The NDVI became saturated at 0.87, or when the LAI value was 6. Across all crop growth stages, eco-sites and seasons, LAI increased rapidly with raising NDVI, while RVI and LAI was more linearly related (R^2^ = 0.80; *p* < 0.01) (Figure 2B), and the effect of saturation was not obvious. NDVI and RVI explained 67% and 70% (*p* < 0.01) of LAI variability at Feekes growth stages 4–7, 79% and 78% (*p* < 0.01) at stages 8–10, respectively (Table 4). 

**Figure 3:**
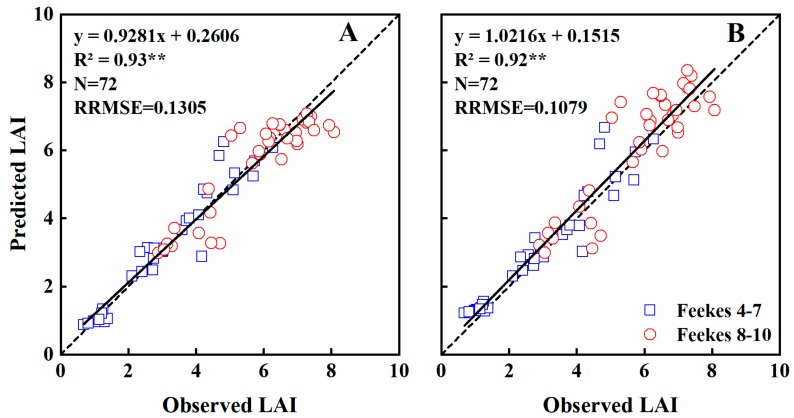
The relationships between observed and predicted LAI values of wheat varieties based on NDVI (**A**) and RVI (**B**) models in the Experiment 4 during vegetation growth period. The dotted line is inclined at 45° to the axes.

The duration model was a good alternative for estimating LAI. The general models across growth stages were validated using a separate dataset. These two models performed similarly (R^2^ ≥ 0.92, RRMSE < 0.14 for both) (Table 5). The coefficients of determination were significant at *p* = 0.01, with most of the scatter plots located close to the 1:1 line (Figure 3).

In all, 96% and 91% of LAI variability was explained by the NDVI and RVI models during Feekes growth stages 4–7 (RRMSE = 0.1426 and 0.1630, respectively), whereas only 77% was explained by each vegetation indices during stages 8–10 (RRMSE = 0.1094 and 0.1264, respectively) (Table 5). 

### 3.3. Estimating Aboveground Dry Matter of Leaves

**Figure 4:**
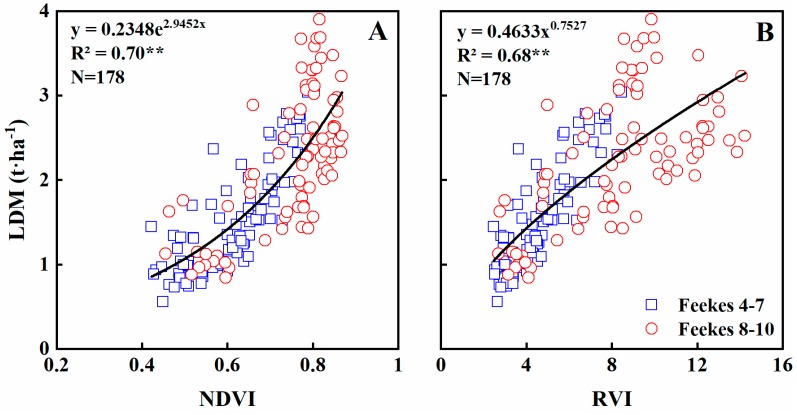
Quantitative relationships of NDVI (**A**) and RVI (**B**) to LDM in wheat varieties under varied N rates across all growth stages.

Leaf dry matter (LDM) is an important indicator of crop growth. Across the entire growth stages, NDVI explained 70% of the LDM variability (Figure 4A). The model showed that LDM increased with raising NDVI, slowly during early stages but then progressively faster. The NDVI became saturated at ~0.87, corresponding to an LDM of ~3 t·ha^−1^. RVI explained 68% of the variability in LDM (Figure 4B), but the saturation effect was not obvious. NDVI and RVI explained 67% and 73% of the LDM variability at Feekes growth stages 4–7, but only 52–54% at stages 8–10 (Table 4).

**Figure 5:**
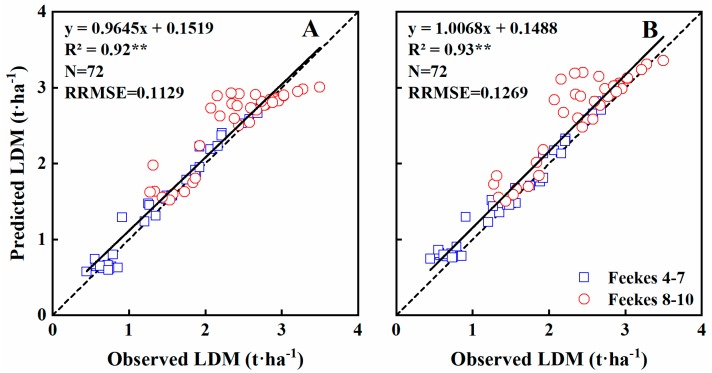
The relationships between observed and predicted LDM values of wheat varieties based on NDVI (**A**) and RVI (**B**) models in the Experiment 4 during vegetation growth period. The dotted line is inclined at 45° to the axes.

The duration model offered a good alternative for estimating LDM. The NDVI and RVI performed similarly, explaining 92% and 93% (*p* < 0.01) of the variability in the LDM (RRMSE = 0.1129, 0.1269), respectively, with most of the scatter plots were close to the 1:1 line (Table 5; Figure 5). At Feekes growth stages 4–7, 94% and 96% of LDM variability was explained by the NDVI and RVI models, with RRMSE values of 0.1491 and 0.1679, respectively. In contrast, at Feekes growth stages 8–10, only 62% and 67% of the LDM variability was explained by the NDVI and RVI models, with RRMSE values of 0.0640 and 0.0730, respectively (Table 5). 

### 3.4. Estimating N Concentration of Leaves

**Figure 6:**
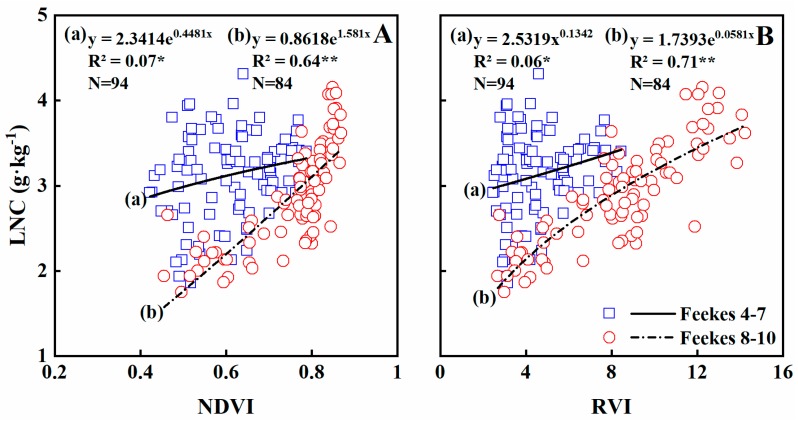
Quantitative relationships of NDVI (**A**) and RVI (**B**) to LNC in wheat varieties under varied N rates across all growth stages.

N concentration is an important nutritional indicator related directly to crop growth status. In-season monitoring of N content is essential to optimize N fertilizer and, thus, reduce environmental risks linked to excess N inputs. In this study, NDVI and RVI were weakly related to LNC, and the relationship was exponential, with R^2^ = 0.10 and 0.12 (*p* < 0.05), respectively (Table 4). Moreover, NDVI and RVI were weakly related to LNC at early stages, explaining 7% and 6% of the variability in LNC at Feekes growth stages 4–7 (Table 4; Figure 6). The R^2^ value was significantly improved at later growth stages, explaining 64% and 71% of the variability in LNC during stages 8–10 (Table 4; Figure 6).

**Figure 7:**
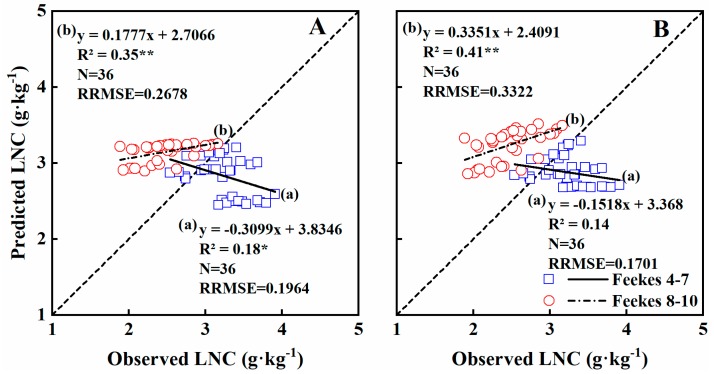
The relationships between observed and predicted LNC values of wheat varieties based on NDVI (**A**) and RVI (**B**) models in the Experiment 4 during vegetation growth period. The dotted line is inclined at 45° to the axes.

The validation results indicated that 27% and 20% of the variability in LNC were explained by NDVI and RVI across growth stages, respectively, with similar RRMSE values (Table 5). At Feekes growth stages 4–7, 18%, and 14% of the variability was explained by NDVI and RVI, with RRMSE values of 0.1964 and 0.1701, respectively (Table 5; Figure 7). At stages 8–10, 35% and 41% was explained by the two models, with RRMSE values of 0.2678 and 0.3322, respectively (Table 5; Figure 7). Thus, stages 8–10 explained more of the variability in leaf N concentration, but with higher RRMSEs. 

### 3.5. Estimating N Accumulation of Leaves

**Figure 8:**
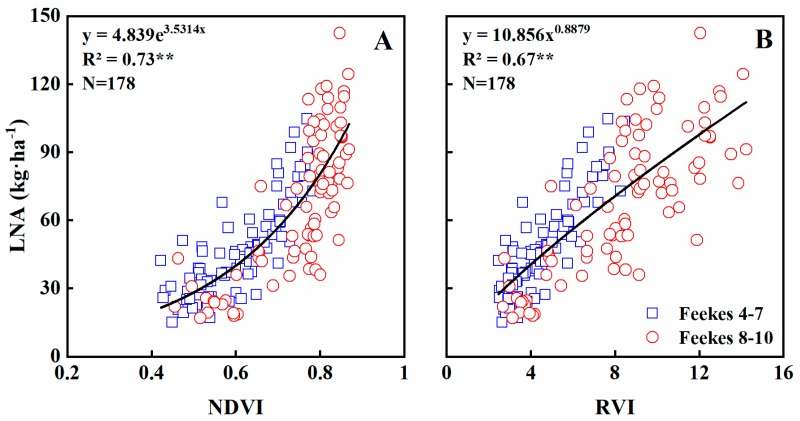
Quantitative relationships of NDVI (**A**) and RVI (**B**) to LNA in wheat varieties under varied N rates across all growth stages.

Leaf N accumulation (LNA) significantly influences grain yield and quality. Furthermore, LNA provides integrated information on N content and LDM and thus is an important parameter to consider when monitoring N nutrition and growth status. In this study, LNA was estimated using the GreenSeeker sensor dataset across all growth stages. NDVI was exponentially related to LNA (R^2^ = 0.73) (Figure 8A) and Feekes growth stages 4–7 and 8–10 explained 68% and 75% of its variability, respectively (Table 4). NDVI became saturated at ~0.87, or when LNA reached ~90 kg·ha^−1^ (Figure 8A). RVI explained 67% of the variability, with a strong relationship (Figure 8B). In fact, RVI was more linearly related to N uptake and was not subject to an obvious saturation effect (Figure 8B). Besides, the relationship between RVI and LNA was stronger during Feekes growth stages 4–7 (R^2^ = 0.75) than stages 8–10 (R^2^ = 0.70) (Table 4).

**Figure 9:**
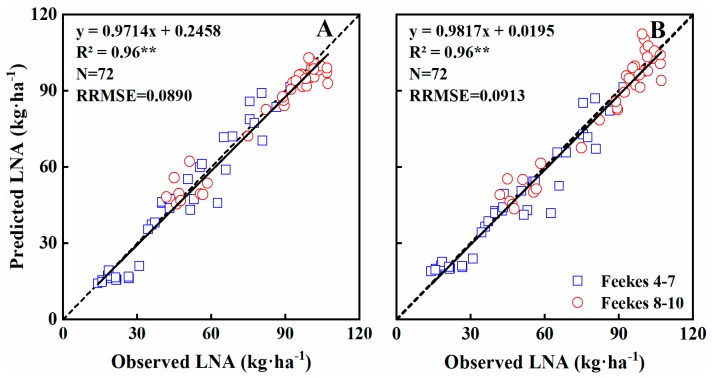
The relationships between observed and predicted LNA values of wheat varieties based on NDVI (**A**) and RVI (**B**) models in the Experiment 4 during vegetation growth period. The dotted line is inclined at 45°to the axes.

Validation results indicated that both NDVI and RVI models explained 96% of the variability in LNA (RRMSE = 0.0890 and 0.0913), across all growth stages (Table 5; Figure 9), 93% and 92% during Feekes growth stages 4–7 (RRMSE = 0.1392 and 0.1395) but only 73% and 75% during stages 8–10 (RRMSE = 0.0953 and 0.0979) (Table 5). Thus, Feekes growth stages 4–7 explained more variability in LNA, but with higher RRMSEs. 

### 3.6. Relationship between Vegetation Index and Relative Grain Yield

**Figure 10:**
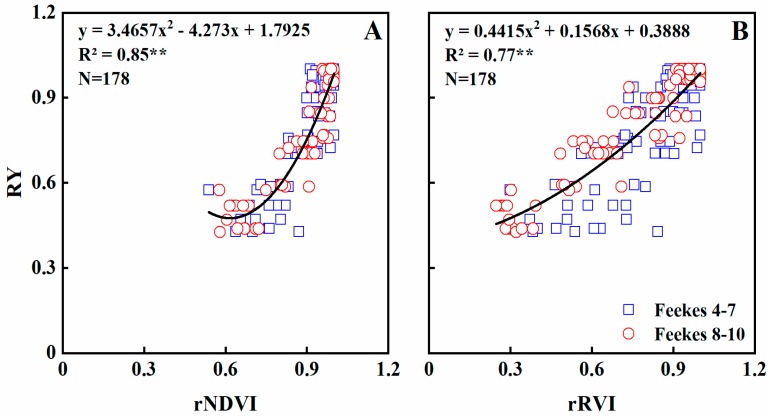
Quantitative relationships of rNDVI (**A**) and rRVI (**B**) to RY in wheat varieties under varied N rates across all growth stages.

Based on the relationship between NDVI values and grain yield of winter wheat, we attempted to establish a regression model. In this study, the newly normalized vegetation indices rNDVI and and rRVI explained 71–81% of the variability in the relative yield (RY) at Feekes growth stages 4–7. Both rNDVI and rRVI explained 90% of the variability at Feekes growth stages 8–10 (Table 6). Across whole growth stages, rNDVI and rRVI explained 77–85% of the variability in RY (Table 6; Figure 10).

**Figure 11:**
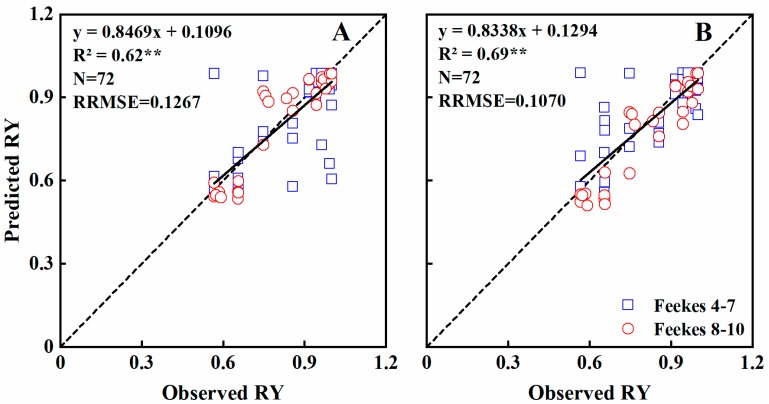
The relationships between observed and predicted RY values of wheat varieties based on rNDVI (**A**) and rRVI (**B**) models in the Experiment 4 during vegetation growth period. The dotted line is inclined at 45° to the axes.

The validation results indicated that the rNDVI and rRVI models explained 62% and 69% of the variability in the relative yield across growth stages (RRMSE = 0.1267 and 0.1070), respectively (Table 7; Figure 11). Only 40% and 50% of the variability were explained by the rNDVI and rRVI models at Feekes growth stages 4–7 (RRMSE = 0.1618 and 0.1334), while the corresponding values for stages 8–10 were 89% and 90% (RRMSE = 0.0720 and 0.0779) (Table 7).

## 4. Discussion

Crop growth parameters, such as LAI, LDM, LNC and LNA, can properly reflect crop growth status and provide data basis for site-specific crop management. In contrast to conventional destructive sampling, remote sensing technology has opened up a new approach to obtain crop growth information due to its fast and non-destructive characteristics. The GreenSeeker active canopy sensor offers a good alternative for acquiring crop growth information with its advantages of simple operation and quick measurement. 

Based on the experimental data of this study, a strong exponential relationship (R^2^ = 0.80) was found between NDVI and LAI across all seasons and growth stages, these results were in agreement with Richardson et al. [39], who used canopy hyper-spectral data to simulate the GreenSeeker sensor, and found the exponential equation was equally good for estimating the LAI with NDVI (R^2^ = 0.83) across the entire growth stages. Goswami et al. [40] found NDVI correlated strongly with LAI (R^2^ = 0.70) but showed saturated when LAI > 2. In this study, the NDVI became saturated similarly when the LAI exceeded a critical value of 6, despite the LAI value induced NDVI to become saturated differs due to different varieties and eco-sites. A logarithmic relationship between RVI and LAI was found in wheat using satellite imagery [41]. In this study, RVI was more linearly related to LAI, this may be caused by the differences in radiation transfer characteristic between two distinct platforms. In all, both NDVI and RVI had robust relationships with LAI. 

Many studies focused on the relationship between aboveground biomass and NDVI or RVI [42,43,44], while few defined the relationship between leaf biomass and these two indices. Our results showed that NDVI and RVI are closely related to LDM. This can be explained by the fact that GreenSeeker is a canopy sensor and is mainly used to sense the upper canopy layer [45], which is better represented by LDM. The NDVI showed saturated at Feekes 8–10 when the LDM was greater than ~3 t·ha^−1^, while the saturated phenomenon was not obvious in RVI. However, the relationship between RVI and LDM became more scattered at later growth stages, which was similar to previous studies [26,46].

For monitoring LNC, GreenSeeker sensor showed a relatively poor performance at early growth stages (R^2^ = 0.06−0.07), while the R^2^ values greatly increased at Feekes 8–10 (R^2^ = 0.64−0.71). Cao et al. [43] also found that at early growth stages (e.g., panicle initiation and stem elongation), the R^2^ values between N concentration of rice plants and vegetation indices were in the range of only 0.03–0.12, but the performances of these indices improved after heading stage (R^2^ = 0.28−0.36). Some related studies have also confirmed this phenomenon [26,47]. Basyouni et al. [25] considered that GreenSeeker readings were less correlated with leaf N concentration at early stages due to plants small size and background noise. These factors demonstrate the challenges in obtaining accurate estimates of crop N concentration using canopy sensors. Moreover, despite the influence of soil background during the early growth stages of plants, the biomass of plants dominates canopy reflectance, due to its faster production than N uptake before heading stage [13,48,49], which increases the difficulty of monitoring N concentration. Studies using hyperspectral remote sensing showed preferable performance in estimating plant N concentration. Using a handheld hyperspectral sensor, Daniela et al. [50] proposed a vegetation index that is able to predict N concentration (R^2^ = 0.65) in rice crops. Based on aerial hyperspectral remote sensing, Cilia et al. [51] found an integrated index, Modified Chlorophyll Absorption Ratio Index/Modified Triangular Vegetation Index 2 (MCARI/MTVI2), performed well in estimating maize plant N concentration (R^2^ = 0.59) and NNI (R^2^ = 0.70). In all, new approaches and further studies are needed to develop reliable models for estimating N concentration of crops. 

Contrary to LNC, LNA showed strong relationship with vegetation indices (VIs) across entire growth stages (R^2^ = 0.67−0.73), and the validation results were acceptable (RRMSE = 8.9−9.1%). Relevant studies also showed that VIs can be used for monitoring LNA [43,47], implying the possibility of monitoring crop nitrogen status using a portable canopy sensor. However, the sensitivity of NDVI decreased at Feekes growth stages 8–10, due to the saturation effect when LNA reached ~90 kg·ha^−1^, corresponding to the LDM of ~3 t·ha^−1^. This also indicates that the saturation effect of NDVI in predicting LNA is mainly caused by the excessive LDM. Nguy-Robertson et al. [52] found that NDVI was most sensitive to LAI below 2, while RVI was most sensitive to LAI above 2, they suggest combining VIs to benefit from different sensitivities of VIs along crop growth stages. This may be the way to avoid the saturation effect, further studies are needed to evaluate the potential of this method in monitoring LDM and LNA.

Previous studies have shown the feasibility of estimating grain yield with remote sensing data in wheat, rice, barely, maize, and soybean [37,53,54,55]. Liu et al. [37] analyzed the quantitative relationship between rice grain yield and canopy NDVI at key growth stages, and the R^2^ values ranged from 0.56–0.62. Feng et al. [54] also found that the correlation coefficient between NDVI and wheat grain yield was in the range of 0.31–0.82 from jointing to filling stages. However, these prediction models varied widely due to different cultivars and eco-sites. In this study, the newly normalized vegetation indices rNDVI and rRVI were calculated to minimize the impact of the above situation. Preferable performance was obtained according to the analysis, rNDVI and rRVI showed strong relationship with RY (R^2^ = 0.77−0.85), and the accuracy of the models at Feekes 8–10 (R^2^ = 0.9) were better than Feekes 4–7 (R^2^ = 0.71−0.81). Freeman et al. [56] found that the yield was most reliably estimated using the NDVI value at booting stage, other estimation models were also developed at heading and filling stages [54,57,58]. These findings are basically consistent with the results of this study. Futhermore, validation of the models at Feekes 8–10 using a separate experiment dataset showed that the R^2^ value were both >0.89 and RRMSEs were both <7.8% (Table 7), which indicated that the performance of the yield prediction models based on newly normalized vegetation indices (rNDVI and rRVI) is better than previous studies [32,37,54,59]. In general, the universality of yield estimation model is susceptible to various factors, such as crop varieties, plant densities, fertilizers and water conditions, these factors should be considered comprehensively to improve the reliability and practicability of model in the future.

No matter LAI, LDM, or LNA, the duration models of these growth indices showed saturated at later growth stages, and the saturation phenomenon mainly appeared in NDVI based models. According to some studies, visible light has a low transmittance through leaves and, therefore, only detects the characteristics of the top layers of the crop canopy after canopy closure, whereas NIR light has a higher transmittance and can thus detect leaves below the top layers of the canopy [13]. Vegetation index NDVI, using NIR and red reflectance, will only increase slightly after a large increase in NIR reflectance, resulting in a saturation effect [55]. The normalization effect embedded in the calculation of NDVI also contributes to the potential saturation of this index; this can be avoided to some extent by using a vegetation index (NIR/R) [42], as demonstrated by this study. Red-edge-based indices, due to the similar light transmittance characteristics of the red-edge wavelength and NIR bands, can reduce the saturation effects in some degree [42]. Our results suggest that the performance of the GreenSeeker sensor may be further improved by adding a red-edge band.

Additionally, only five cultivars with N treatments were used in this study, and the sampling frequency and interval are also limited. Additionally, experiment sites were restricted in Jiangsu province, China, where cultivars and environments are relatively analogous. These factors affected the universality of monitoring models, further studies with diverse cultivars, nitrogen levels, and eco-sites are needed to solve these problems, and to improve the performance of the models.

## 5. Conclusions

Active canopy sensors are highly suitable for real-time diagnosing crop growth. In this study, the performance of GreenSeeker in estimating growth indices and yield potential of winter wheat was evaluated in Jiangsu province, China. The analysis indicated that NDVI and RVI performed well in estimating LAI, LDM, and LNA across entire growth stages, with highest R^2^ values reached 0.8, 0.7, and 0.73, respectively. The performance of GreenSeeker in monitoring LNC was relatively poor at Feekes 4–7 (R^2^ = 0.06−0.07) but greatly improved at later growth stages (R^2^ = 0.64−0.71). The newly normalized vegetation indices (rNDVI and rRVI) were developed to predict grain yield and correlated well with relative yield, with R^2^ values reached 0.9 and RRMSE below 0.078 at Feekes 8–10. In summary, Greenseeker and sensor-based models for monitoring growth status and predicting grain yield have the advantages of simple structure and easy operation in site-specific crop management, and this study enriches the theoretical basis and technical approach of real-time crop growth information acquisition for precision agriculture. Further research with multiple cultivars and eco-sites should be conducted to improve these models and enhance the practicability of GreenSeeker in field production.

## Figures and Tables

**Table 1:** Basic information about four field experiments.

Experiment NO.	Location	Variety	N Rate (kg·ha^−1^)	Sampling Stage/Date	Soil Characteristics
Experiment 1 2013–2014	Rugao (32°27′ N, 120°76′ E)	Xumai-30 Ningmai-13	N0 (0) N1 (75) N2 (150) N3 (225) N4 (300)	Feekes 4 (14 February) Feekes 5 (24 February) Feekes 6 (9 March) Feekes 7 (15 March) Feekes 8 (27 March) Feekes 9 (4 April) Feekes 10 (10 April) Feekes 10.2 (15 April)	Soil type = Loam soil Soil pH = 6.4 OM = 23.15 g·kg^−1^ Total N = 1.45 g·kg^−1^ Available P = 47.10 mg·g^−1^ Available K = 112.50 mg·g^−1^
Experiment 2 2013–2014	Xuzhou (34°48′ N, 117°13′ E)	Xumai-30 Jimai-13	N0 (0) N1 (90) N2 (180) N3 (270) N4 (375)	Feekes 4 (4 March) Feekes 6 (20 March) Feekes 7 (2 April) Feekes 10 (12 April) Feekes 10.2 (24 April)	Soil type = Loam soil Soil pH = 6.5 OM = 24.50 g·kg^−1^ Total N = 1.35 g·kg^−1^ Available P = 45.10 mg·g^−1^ Available K = 116.00 mg·g^−1^
Experiment 3 2014–2015	Huai’an (33°60′ N, 118°88′ E)	Ningmai-13 Yangfumai-4 Huaimai-20	N0 (0) N1 (120) N2 (225) N3 (330)	Feekes 5 (16 March) Feekes 7 (31 March) Feekes 10 (12 April) Feekes 10.2 (20 April)	Soil type = Loam soil Soil pH = 6.3 OM = 22.35 g·kg^−1^ Total N = 1.30 g·kg^−1^ Available P = 46.20 mg·g^−1^ Available K = 110.50 mg·g^−1^
Experiment 4 2014–2015	Rugao (32°27′ N, 120°76′ E)	Ningmai-13 Yangfumai-4 Huaimai-20	N0 (0) N1 (120) N2 (225) N3 (330)	Feekes 4 (9 February) Feekes 6 (8 March) Feekes 7 (19 March) Feekes 8 (28 March) Feekes 10 (8 April) Feekes 10.3 (18 April)	Soil type = Loam soil Soil pH = 6.4 OM = 23.55 g·kg^−1^ Total N = 1.55 g·kg^−1^ Available P = 44.80 mg·g^−1^ Available K = 110.50 mg·g^−1^

**Table 2:** Statistical analysis of LAI, LDM, LNC, and LNA at different growth stages across eco-sites and seasons.

Parameter	Growth Stage	Calibration Data				Validation Data			
		N	Range	SD	CV (%)	N	Range	SD	CV (%)
LAI	Feekes 4–7	94	1.16–6.63	1.29	43.02	36	0.68–5.74	1.56	54.82
	Feekes 8–10	84	1.26–9.47	2.00	38.97	36	3.16–8.34	1.41	22.74
	All stages	178	1.16–9.47	1.97	49.24	72	0.68–8.34	2.25	49.60
LDM (t·ha^−1^)	Feekes 4–7	94	0.56–3.04	0.60	39.44	36	0.45–2.11	0.47	39.37
	Feekes 8–10	84	0.84–3.90	0.78	34.16	36	1.24–3.50	0.56	25.56
	All stages	178	0.56–3.90	0.79	41.86	72	0.45–3.50	0.72	42.75
LNC (g·kg^−1^)	Feekes 4–7	94	18.54–43.12	0.50	15.97	36	2.54–3.91	0.32	9.88
	Feekes 8–10	84	17.52–41.53	0.60	20.66	36	1.89–3.16	0.33	13.46
	All stages	178	17.52–43.12	0.56	18.56	72	1.89–3.91	0.51	17.81
LNA (kg·ha^−1^)	Feekes 4–7	94	14.99–104.56	21.00	43.92	36	14.12–67.52	14.77	38.77
	Feekes 8–10	84	16.97–142.37	30.71	44.88	36	26.62–99.54	17.93	32.67
	All stages	178	14.99–142.37	28.00	48.66	72	14.12–99.54	18.44	39.67

LAI: leaf area index; LDM: leaf dry matter; LNC: leaf nitrogen concentration; LNA: leaf nitrogen accumulation. N, sampling number; Mean: average value; SD: standard deviation; CV: coefficient of variation.

**Table 3:** Variance analysis of LAI, LDM, LNC, and LNA in different varieties (V), years (Y), and N rates (N).

Parameter	df	LAI		LDM (t·ha^−1^)		LNC (g·kg^−1^)		LNA (kg·ha^−1^)	
		MS	F-Value	MS	F-Value	MS	F-Value	MS	F-Value
V	4	8.39	2.21	1.28	2.11	0.14	0.43	649	0.81
Y	1	6.74	1.73	10.71 **	19.03	0.11	0.34	5767 **	7.59
N	4	53.58 **	19.44	7.12 **	15.16	8.20 **	61.74	16,595 **	39.42
V × Y	-	-	-	-	-	-	-	-	-
V × N	14	1.02	0.3	0.07	0.17	0.03	0.22	46.55	0.13
Y × N	3	3.24	1.19	0.11	0.26	0.06	0.43	57.11	0.16
V × Y × N	-	-	-	-	-	-	-	-	-

LAI: leaf area index; LDM: leaf dry matter; LNC: leaf nitrogen concentration; LNA: leaf nitrogen accumulation; df: degree of freedom; MS: mean square; Wheat varieties include Xumai-30, Ningmai-13, Jimai-13, Yangfumai-4 and Huaimai-20; Years are 2013 and 2014; N rates include N(0)–N(4); ** F-test: statistical significance at the 0.01 probability level. Different wheat varieties were used in 2013 and 2014, thus V × Y and V × Y × N cannot be evaluated.

**Table 4:** Coefficient of determination between NDVI, RVI and wheat agronomic parameters at different stages in experimental fields across site-years.

Agronomic Parameter	Feekes Growth Stage	NDVI		RVI	
		Equation	R^2^	Equation	R^2^
LAI	4–7	E	0.67 **	L	0.7 **
	8–10	E	0.79 **	P	0.78 **
	All stages	E	0.8 **	P	0.8 **
LDM (t·ha^−1^)	4–7	E	0.67 **	L	0.73 **
	8–10	E	0.54 **	P	0.52 **
	All stages	E	0.7 **	P	0.68 **
LNC (g·kg^−1^)	4–7	E	0.07 *	P	0.06 *
	8–10	E	0.64 **	E	0.71 **
	All stages	E	0.1 *	E	0.12 *
LNA (kg·ha^−1^)	4–7	E	0.68 **	L	0.75 **
	8–10	E	0.69 **	P	0.7 **
	All stages	E	0.73 **	P	0.67 **

LAI: leaf area index; LDM: leaf dry matter; LNC: leaf nitrogen concentration; LNA: leaf nitrogen accumulation. E: exponential equation; L: linear equation; P: power equation; Q: quadratic equation. * F-test: statistical significance at the 0.05 probability level. ** F-test: statistical significance at the 0.01 probability level.

**Table 5:** Validation of the GreenSeeker indices for estimating LAI, LDM, LNC, and LNA at different wheat growth stages.

Agronomic Parameter	Feekes Growth Stage	NDVI			RVI		
		R^2^	RRMSE	RE(%)	R^2^	RRMSE	RE(%)
LAI	4–7	0.96 **	0.1426	10.13	0.91 **	0.1630	11.07
	8–10	0.77 **	0.1094	10.32	0.77 **	0.1264	13.06
	All stages	0.93 **	0.1305	9.95	0.92 **	0.1079	9.08
LDM (t·ha^−1^)	4–7	0.94 **	0.1491	11.06	0.96 **	0.1679	13.28
	8–10	0.62 **	0.0640	9.47	0.67 **	0.0730	12.42
	All stages	0.92 **	0.1129	12.65	0.93 **	0.1269	14.14
LNC (g·kg^−1^)	4–7	0.18 *	0.1964	12.27	0.14	0.1701	12.37
	8–10	0.35 **	0.2678	24.84	0.41 **	0.3322	29.90
	All stages	0.27 **	0.2401	21.74	0.20 **	0.2358	22.64
LNA (kg·ha^−1^)	4–7	0.93 **	0.1392	10.31	0.92 **	0.1395	10.05
	8–10	0.73 **	0.0953	12.13	0.75 **	0.0979	11.14
	All stages	0.96 **	0.0890	8.48	0.96 **	0.0913	8.96

LAI: leaf area index; LDM: leaf dry matter; LNC: leaf nitrogen concentration; LNA: leaf nitrogen accumulation. * F-test statistical significance at the 0.05 probability level. ** F-test statistical significance at the 0.01 probability level.

**Table 6:** Coefficient of determination between rNDVI, rRVI, and wheat yield at different stages in experimental fields across site-years.

GROWTH Stage	rNDVI		rRVI	
	Regression Equation	R^2^	Regression Equation	R^2^
Feekes 4–7	RY = 3.14rNDVI^2^ − 3.69rNDVI + 1.54	0.81 **	RY = 0.60rRVI^2^ + 0.002rRVI + 0.38	0.71 **
Feekes 8–10	RY = 3.87rNDVI^2^ − 4.98rNDVI + 2.08	0.9 **	RY = 0.72rRVI + 0.27	0.9 **
All stages	RY = 3.47rNDVI^2^ − 4.27rNDVI + 1.79	0.85 **	RY = 0.44rRVI^2^ + 0.16rRVI + 0.39	0.77 **

** F-test statistical significance at the 0.01 probability level.

**Table 7:** Validation results of the GreenSeeker indices for estimating wheat yield at different growth stages.

Growth Stage	rNDVI			rRVI		
	R^2^	RRMSE	RE (%)	R^2^	RRMSE	RE (%)
Feekes 4–7	0.4 **	0.1618	10.55	0.5 **	0.1334	10.54
Feekes 8–10	0.89 **	0.0720	6.42	0.9 **	0.0779	5.29
All stages	0.62 **	0.1267	5.93	0.69 **	0.1070	6.29

** F-test statistical significance at the 0.01 probability level.
